# Supplementary material for: Usability, safety and tolerability of CUE1 vibrotactile device as promising therapeutic approach in orthostatic tremor
Source: Clin Park Relat Disord. 2025 Aug 4;13:100379. doi: 10.1016/j.prdoa.2025.100379 (PMC12345306; doi:10.1016/j.prdoa.2025.100379)
Supplement: Supplementary Data 1 [file mmc1.docx]

# **Supplementary Material Files**

## **Supplementary Material A.** The CUE1 device.

The CUE1 device is designed to provide non-invasive support for both motor and non-motor symptoms of Parkinson's. It is a compact device, with dimensions of 40 mm in diameter, 11 mm in height, and a weight of 17 g. The device attaches to the sternum using dermatologically tested adhesive patches that are waterproof, with each patch capable of remaining in place for up to 14 days without replacement. While the CUE1 itself is water-resistant, it is not fully waterproof and should be removed prior to exposure to water, such as during showering. The CUE1 utilizes a silent motor to deliver vibrotactile stimulation, with a stimulation pattern developed through user testing in people with Parkinson’s and Primary Orthostatic Tremor. Although initial clinical trials have been conducted in people with Parkinson’s, clinical trials are needed to assess the mechanisms, feasibility, safety, tolerability, and efficacy of CUE1 in people with Primary Orthostatic Tremor.


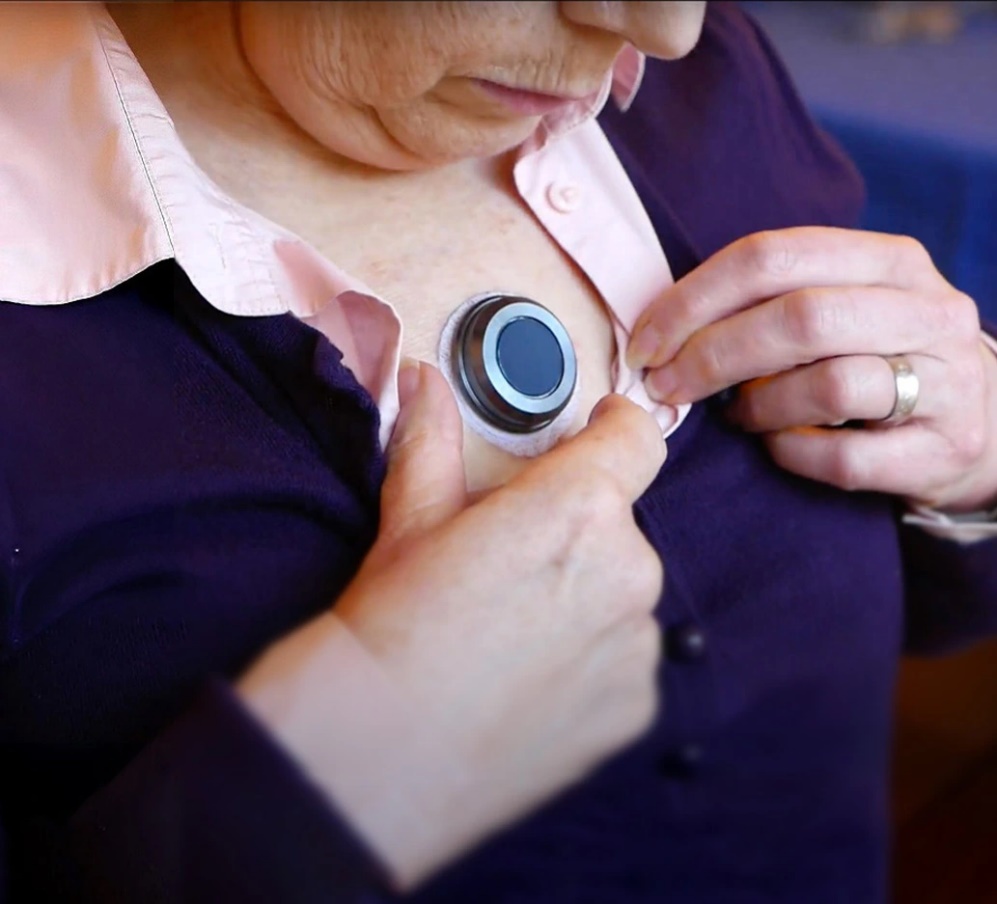


## **Supplementary Material B.** Flow of participants through this study.

Excluded (n= 82):

- Live far from research site (n= 76)
- No official confirmation of orthostatic tremor available (n= 5)
- Include other diagnosis affecting balance and walking (n=1)

Intervention 2:

Wear the CUE1 for 8 hours daily, starting in the morning, over a period of 9 weeks.

Follow up assessment (n= 10)

Intervention 1:

Wear the CUE1 for 20 minutes on the day of the baseline assessment.

Baseline assessment 1 (n= 10)

Baseline assessment 2 (n= 10)

Recruited (n= 10)

Assessed for eligibility (n= 92)

## **Supplementary Material C.** Participants’ feedback on receiving CUE1 intervention.

| **Participants’ Satisfaction with CUE1** | **Participants**  **(n = 10)** |
| --- | --- |
| How helpful did you find the CUE1 device in alleviating your symptoms?^a^ | 1.55 (0.93) |
| How satisfied were you with using the CUE1 device?^a^ | 3.00  (1.00, 3.00) |
| If given the option, how likely are you to continue using the CUE1 device?^a^ | 3.00  (3.00, 3.00) |
| How easy was it for you to use the CUE1 device in the recommended body position (e.g., sternum)?^a^ | 3.00  (3.00, 3.00) |
| How easy was it for you to apply the adhesive patches provided with the CUE1 device to the recommended body position (e.g., sternum)?^a^ | 3.00  (3.00, 3.00) |
| How likely are you to recommend the CUE1 device to others with Orthostatic Tremor who experience similar symptoms to yours?^a^ | 3.00  (2.00, 3.00) |

Data are mean (SD) or median (IQR). N, number; %, percentage; SD= Standard deviation. IQR= 1^st^ and 3^rd^ Interquartile. ^a^Responses are shown on a 5-point Likert scale, where 0 indicates the lowest (e.g., not at all) rating and 4 indicates the highest (e.g., extremely) rating.
